# Complex Inheritance of Rare Missense Variants in *PAK2, TAP2*, and *PLCL1* Genes in a Consanguineous Arab Family With Multiple Autoimmune Diseases Including Celiac Disease

**DOI:** 10.3389/fped.2022.895298

**Published:** 2022-06-15

**Authors:** Arwa Mastoor Alharthi, Babajan Banaganapalli, Sabah M. Hassan, Omran Rashidi, Bandar Ali Al-Shehri, Meshari A. Alaifan, Bakr H. Alhussaini, Hadeel A. Alsufyani, Kawthar Saad Alghamdi, Khalda Khalid Nasser, Yagoub Bin-Taleb, Ramu Elango, Noor Ahmad Shaik, Omar I. Saadah

**Affiliations:** ^1^Department of Biological Sciences, Faculty of Science, King Abdulaziz University, Jeddah, Saudi Arabia; ^2^Princess Al-Jawhara Al-Brahim Center of Excellence in Research of Hereditary Disorders, King Abdulaziz University, Jeddah, Saudi Arabia; ^3^Department of Genetic Medicine, Faculty of Medicine, King Abdulaziz University, Jeddah, Saudi Arabia; ^4^Princess Najla Bint Saud Al-Saud Center for Excellence Research in Biotechnology, King Abdulaziz University, Jeddah, Saudi Arabia; ^5^Saudi Ajal for Health Services, Riyadh, Saudi Arabia; ^6^Department of Pediatrics, Faculty of Medicine, King Abdulaziz University, Jeddah, Saudi Arabia; ^7^Pediatric Gastroenterology Unit, Department of Paediatrics, King Abdulaziz University Hospital, Jeddah, Saudi Arabia; ^8^Department of Medical Physiology, Faculty of Medicine, King Abdulaziz University Hospital, Jeddah, Saudi Arabia; ^9^Department of Biology, Faculty of Science, Hafar Al-Batin University, Hafar Al-Batin, Saudi Arabia; ^10^Department of Medical Laboratory Technology, Faculty of Applied Medical Sciences, King Abdulaziz University, Jeddah, Saudi Arabia; ^11^Centre of Artificial Intelligence in Precision Medicine, King Abdulaziz University, Jeddah, Saudi Arabia

**Keywords:** complex inheritance, multiple autoimmune diseases, celiac disease, WES, consanguineous

## Abstract

**Background:**

Autoimmune diseases (AIDs) share a common molecular etiology and often present overlapping clinical presentations. Thus, this study aims to explore the complex molecular basis of AID by whole exome sequencing and computational biology analysis.

**Methods:**

Molecular screening of the consanguineous AID family and the computational biology characterization of the potential variants were performed. The potential variants were searched against the exome data of 100 healthy individuals and 30 celiac disease patients.

**Result:**

A complex inheritance pattern of *PAK2* (V43A), *TAP2* (F468Y), and *PLCL1* (V473I) genetic variants was observed in the three probands of the AID family. The *PAK2* variant (V43A) is a novel one, but *TAP2* (F468Y) and *PLCL1* (V473I) variants are extremely rare in local Arab (SGHP and GME) and global (gnomAD) databases. All these variants were localized in functional domains, except for the *PAK2* variant (V43A) and were predicted to alter the structural (secondary structure elements, folding, active site confirmation, stability, and solvent accessibility) and functional (gene expression) features. Therefore, it is reasonable to postulate that the dysregulation of *PAK2*, *TAP2*, and *PLCL1 genes* is likely to elicit autoimmune reactions by altering antigen processing and presentation, T cell receptor signaling, and immunodeficiency pathways.

**Conclusion:**

Our findings highlight the importance of exploring the alternate inheritance patterns in families presenting complex autoimmune diseases, where classical genetic models often fail to explain their molecular basis. These findings may have potential implications for developing personalized therapies for complex disease patients.

## Introduction

Autoimmune diseases (AIDs) are triggered by the failure of the immune system to distinguish between self and non-self-antigens. This group of diseases are a significant cause of a large share of morbidity, early mortality, and disability worldwide. However, their epidemiology differs depending on ethnic origin and locations (1).

There are more than 100 distinct autoimmune disorders that are organ-specific or affect multiple organs. For example, in systemic lupus erythematosus (SLE), the immune system attacks its own tissues (2) and affects many organs; autoimmune thyroid disorders (AiT) damage the thyroid glands (3); and type 1 diabetes (T1DM) damages the pancreatic beta cells (4). In Celiac disease (CeD), exposure to gluten, a small peptide found in barley, wheat, and rye, results in autoantibodies resulting in intestinal damage (5). The prevalence rate of the autoimmune diseases varies from 1.5 to 11 per 100,000 person-years for SLE (6), 7.3 per 100,000 person-years for T1DM (7), 27 to 273 per 100,000 for AiT (8), and 3.4% for CeD (9).

The causal factors for autoimmune diseases are not well established. However, it is widely believed that complex molecular interactions between genes and environmental factors contributes to the onset and progression of autoimmune diseases. Genome-wide association studies (GWAS) have expanded our understanding of the influence of genetic variations on the susceptibility to different auto-immune diseases (10, 11). These studies have shown that almost all autoimmune and inflammatory diseases share many susceptibility loci in the genome, with the best example being the variants of the class II *HLA* gene, *HLA DRB1* (12). Not only that, SLE and T1DM may have a common underlying etiological pathway involving the *PTPN22* gene. The variant of *PTPN22* is shown to increase the risk of T1DM as well as SLE in the carrier (13). One meta-analysis of multiple GWAS, combined with animal studies showed 18 clusters of major loci that are risk factors for two or more of the autoimmune diseases (14).

Increased risk of developing the second autoimmune disease for an individual with an existing one is already well known (15). The co-occurrence of multiple autoimmune diseases in an individual is very common due to the overlapping genetic architecture and functions. About 25% of individuals with autoimmune disorders are likely to develop one or more autoimmune diseases (16). It is well documented that individuals with celiac disease will manifest other autoimmune disorders more often (∼5%) than healthy people (10). Also, individuals with T1DM and AiT usually test positive for Celiac disease (10). However, these studies were based only on sporadic cases where it is difficult to identify any causal genes (17–22).

When individuals have more than one autoimmune disease, investigating polygenic inheritance may provide clues for elucidating common pathways that could provide therapeutic targets for more than one disease. The advent of powerful high-throughput next-generation sequencing technologies like whole-exome sequencing has provided an opportunity to identify disease candidate genes, novel causal variants in known genes and their inheritance patterns in familial cases of complex diseases (21, 23, 24).

In this study, we applied whole-exome sequencing to a rare consanguineous Saudi family with four segregating autoimmune diseases (CeD, AiT, SLE, and TIDM) to find the causal genes which may explain such disease clustering due to complex aggregation of rare variants in more than one gene.

## Materials and Methods

Overall workflow of the current study represented in Supplementary Figure 1.

### Recruitment of Multi-Autoimmune Diseases Family

This research proposal was approved by the Research Ethics Committee at King Abdulaziz University Hospital, Jeddah (KAUH). The Saudi consanguineous family with three affected individuals (the mother and her two sons) was referred to the gastroenterology clinic at King Abdulaziz University Hospital for Celiac disease diagnosis. In brief, the two siblings were initially diagnosed first with T1DM, followed by AiT and CeD, respectively. Then, subsequent family interview has suggested that the mother is also suffering with symptoms indicative of SLE, AiT, and CeD. The follow-up clinical and laboratory test results, following the well-recognized international diagnostic criteria, has confirmed the diagnosis of CeD, AiT, SLE, and TIDM in mother and her two sons (25, 26). The multi-generation pedigree was constructed based on the interview with the family members. The signed informed consent was obtained from all the family members after explaining them this study protocol. Approximately 3–5 ml of peripheral blood sample from each family member were collected in EDTA tubes for the genetic analysis.

### DNA Extraction

The genomic DNA was isolated and purified using the QIAamp DNA Blood kit and following the manufacturer’s instructions. The purity and concentration of the isolated DNAs were determined with the help of a Nanodrop spectrophotometer (ND-1000 UV–VIS) considering optical density (OD) values at 260/280nm ratio between 1.8 and 2.0. The DNA integrity was tested by running the 5 μl of DNA on a 1% agarose gel.

### Whole Exome Sequencing Analysis

Genomic DNA (100 ng/μl) was used for preparing the fragment library that can be easily sequenced. Exome capture of the protein-coding regions was carried out after the hybridization of the fragmented DNA with biotinylated cRNA library baits with the Agilent exome library V6 kit following the manufacturer’s instructions (21, 27). Targeted DNA was amplified and sequenced using the HiSeq2000 Next Generation Sequencer (Illumina, San Diego, CA). The generated results (in FASTQ format). Sequences were aligned against the human genome reference sequence build 38 (GRCH38.p12) using the Burrows–Wheeler Aligner.^1^ The genome analysis toolkit (GATK) was used for base quality recalibration (28). In each read, the SAM tools were utilized for calling SNPs and short insertion/deletion (29). The alignment sequencing data coverage was ∼100X, which represented 87% of the target regions.

### Variant Filtering and Candidate Gene Selection

Autoimmunity candidate gene lists were collected from different databases (NCBI, GWAS catalog, and celiac gene panels) to confirm their presence in the exome data. The filtration criteria of the Whole Exome Sequencing (WES) data are to include variants if they first passed the sequencing quality filter, mapped to the coding or regulatory region with the minor allele frequency (MAF) < 1.5% in the gnomAD database. Furthermore, the resultant variants were also filtered by their functional relevance to autoimmune diseases in general, and particularly to CeD, AiT, SLE, and TIDM. The WES data of more than 2300 Saudi volunteers, hosted on the Saudi Human Genome Project web server (SHGP^2^), was used to assess the MAF of the filtered variants in the Saudi population and the Great middle Eastern Variome (GME database).

### Sanger Sequencing of Candidate Variant

The potential candidate variants identified from filtration and comparison of WES data among the probands vs healthy family members were further confirmed through Sanger Sequencing. In this regard, we initially designed target-specific oligonucleotide primers with an average of 400–600 bp using the open-source software NCBI Primer Blast (30). All the sequence reads were aligned and annotated against the reference mRNA sequence of the potential candidate variants with the BioEdit^3^ program.

### Protein Interaction and Pathways Enrichment Analysis

The protein-protein interaction (PPI) networks of the candidate genes were generated with the STRING webserver.^4^ For the construction of the interaction network, the minimum required interaction score was set to 0.400 (medium confidence) along with other default settings. Then, Cytoscape 3.8.2 software was used for the PPI network visualization.

### Computational Validation and Functional Analysis

The three candidate genes from our family were further analyzed to explore their possible contributions to the different auto-immune disorders in this family. In this framework, several computational tools and databases were used to carry out functional enrichment annotations and gene expression levels in different organs and tissues.

#### Gene Ontology and Gene Expression Analysis

The functionally enriched key GO terms, including biological processes, molecular function, cellular components, and pathways for all three genes, were analyzed using the Ensembl database.^5^ The candidate genes’ gene expression data were obtained from the Genotype-Tissue Expression (GTEx) project, which is available in Ensembl. The output provides a comprehensive resource of tissue-specific gene expression and regulation in transcripts per million (TPM) for specific genes.

#### Sequence Analysis

The primary sequence information (nucleotide as well as amino acids) of the genes of interest was derived from NCBI GenBank. Sequence analysis like conserved domain identification and superfamily detection has been carried out using the SMART server.^6^ To understand the sequence conservation pattern throughout evolution as well as to establish their phylogenetic relationship with primates (12 selected primates), multiple sequences of the candidate genes spanning the variant location were analyzed using the Ensembl genome browser^7^ and the NGPhyloge web server.^8^

### Protein Structure Prediction and Analysis

To understand the effect of variants (due to candidate rare variants) on the native protein structural conformation, we used the three-dimensional (3D) structure of the protein using the homology modeling approach. In this study, the protein models were predicted using I-Tasser webserver. In the I-Tasser tool, based on the confidence score (C-score), the best structural model was selected. For all the models, energy minimization was done by applying a 1500 step steepest descent algorithm using the Swiss-PDB Viewer. The energy minimized model structure was validated using Ramachandran plot analysis using Zlab.^9^ The DUET web server^10^ was used for predicting the mutated structural conformation. In addition, the PredictProtein web tool^11^ was utilized to discover the change in solvent accessibility and flexibility of the candidate protein in both wild-type and variant conditions.

## Results

### Clinical Presentation of Multiple Autoimmune Diseases in the Family

**FIGURE 1:**
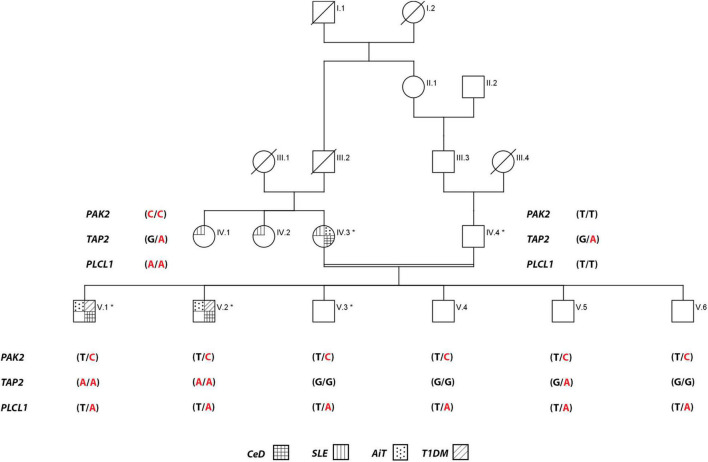
Pedigree of the consanguineous family multiple autoimmune disease. Exome data generated for individuals with an * mark. For *PAK2* gene, T is wild-type, and C is the variant allele**,** *TAP2* gene, G is wild-type, and A is the variant allele, and *PLCL1* gene, T is wild-type, and A is the variant allele.

The proband of one consanguineous Saudi family has self-reported that the family is relatively free from any auto-immune disease except AiT, which is diagnosed in his mother and two of her sisters (Figure 1). Detailed family history and clinical data of the nuclear family revealed that the proband V.1 (23 years old now) was initially diagnosed with AiT (at 10 years of age). After a few months, he was diagnosed with T1DM. At 12 years of age, his family started noticing short stature, weight loss, and a lack of appetite in him. His endoscopy test showed villous atrophy and crypt hyperplasia with intraepithelial lymphocytes, and his tTG antibody test confirmed CeD. The age of diagnosis of T1DM in his younger brother V.2 (21 years old now) was 7 years old. After that, he was diagnosed with AiT. At 15 years of age, he started having the same symptoms as his elder brother; short stature, weight loss, and lack of appetite. His duodenal biopsy confirmed the CeD pathology. At first, Mother (IV.5) was initially diagnosed with SLE, and at the same time, she was also diagnosed with AiT. During the serological screening of the whole family to identify the CeD, the mother (IV.5) was positive with endomysial antibodies. All other members of the family were found to be negative. The mother has reported mild gastrointestinal symptoms. No endoscopy was done on her to confirm the CeD diagnosis. All three affected individuals (including the mother) were put on a gluten-free diet.

### Whole Exome Sequencing: Raw Variant Data and Prioritization

Whole-exome sequencing of five individuals in this family was carried out, including three affected (V.1, V.2, and IV.5), one of the healthy siblings (V.3), and the father (IV.6). The output of the exome data includes approximately 100 K variants per sample (Table 1). We excluded the non-coding (downstream, upstream, intergenic, and intronic variants) and synonymous variants (except the splice sites). The 1000 Genomes Project and gnomAD were used to filter the variant data, excluding variants with MAFs greater than 0.015 (Table 1) (Supplementary Figure 2).

**TABLE 1:** The exome variant yield of the family members.

			Variant filtration results				

Member		Total no. of variants	Zygosity		Coding and regulatory variants	Missense variants	Rare variants (<0.015% frequency)
			Variant status	No. of variants			
IV0.6	Father	101,632	Het	64,816	9445	12,584	1883
			Hom	36,815			
IV0.5	Mother	102,840	Het	67,237	9856	12,724	2156
			Hom	35,602			
V0.1	Affected 1	97,716	Het	56,991	9088	11,933	1809
			Hom	40,724			
V0.2	Affected 2	98,181	Het	59,060	8832	12,079	2510
			Hom	39,120			
V0.3	Normal child	101,401	Het	63,416	9440	12,608	2139
			Hom	37,984			

*Filtering criteria for variants: exonic, unknown, or extremely rare (MAF = <0.015), type (missense, frame-shifts, indels, and splice sites).*

We investigated the exome data of this family to find the variants that show either an autosomal recessive (AR), an autosomal dominant (AD), or compound heterozygous mode of inheritance. In the AR inheritance model, (a) the affected individuals are expected to show the causal allele in homozygote condition, while the healthy members could be either heterozygous carriers or homozygous for the normal allele. (b) In the compound heterozygous inheritance pattern, the affected individuals inherit two different variants (one from each parent) of the same gene, but not the healthy family members. In the AD inheritance model, affected individuals carry one copy of the variant, which is absent in healthy family members. For all inheritance modes, variants with the following scores of variant effect prediction tools of non-deleterious nature (SIFT score < 0.01 or FATHMM-MKL score > 0.5 predictions) were excluded from the analysis. We found 8 variants with the classical AR inheritance pattern, 7 variants in compound heterozygote mode, and 15 variants in the AD pattern. We could not find any single gene whose variation that could explain the inheritance of 4 autoimmune diseases in the family. Genes mapped to these above-mentioned variants were ruled out as potential candidates due to their unrelated functions to any of the autoimmune diseases seen in the family. Therefore, we searched for a complex inheritance of more than one gene variant by searching for the shared rare variants between all affected and healthy individuals in this family (Het and Hom) and ruled out what is seen as homozygous in healthy individuals. The results were 226 variants that are seen in affected individuals as Het and Hom, and only as Het in the healthy. By functional searching, we found 3 rare variants (listed in Table 2) that have been reported to be functionally relevant and are connected by their role in different auto-immune disorders seen in this family. In all affected members, at least one of three gene variants is homozygous, and the other two are heterozygous. The mother carried homozygous for rare variants in *PAK2* (c.128T > C, p. V43A) and *PLCL1* (c.1403T > A, p. V473I) genes, and all family members had only one copy, both affected and unaffected. But she has one copy of a rare variant in the *TAP2* gene (c.1417G > A, p. F468Y). The mother is affected by SLE, AiT, and CeD. Both affected siblings have AIT, T1DM, and CeD. They inherited rare variants in *PAK2* and *PLCL1* (het) from their mother and in *TAP2* genes (Hom) from both parents. None of the healthy members of the family are homozygous for these variants. None of the healthy relatives carries two copies of any of the variants. The existence of these three variants together in an individual, at least one in homozygous condition, explains the complex inheritance of four autoimmune diseases in this family.

**TABLE 2:** List of nucleotide variants from exome data which is found only in the affected individuals with the frequencies of the three rare variants in SHGP, GME, and genomAD.

Gene name	Genomic location	cDNA location	Effect	protein effect	RS ID	Variant frequency (homozygous)	SHGP	GME allele frequency	GenomAD
*PAK2*	3-196,782,774	c.128T > C	missense_ variant	p.Val43Ala	Novel	Novel	Novel	Novel	Novel
*PLCL1*	2-198,084,920	c.1403T > A	missense_ variant	p.Phe468Tyr	rs754994541	0.0004	0.0098	0.0025	2.040850e^–05^
*TAP2*	6-32,830,662	c.1417G > A	missense_ variant	p.Val473Ile	rs765178638	0.002	0.0106	0.0016	4.148104e^–05^

### Validation of Family-Specific Variants Using Sanger Sequencing

**FIGURE 2:**
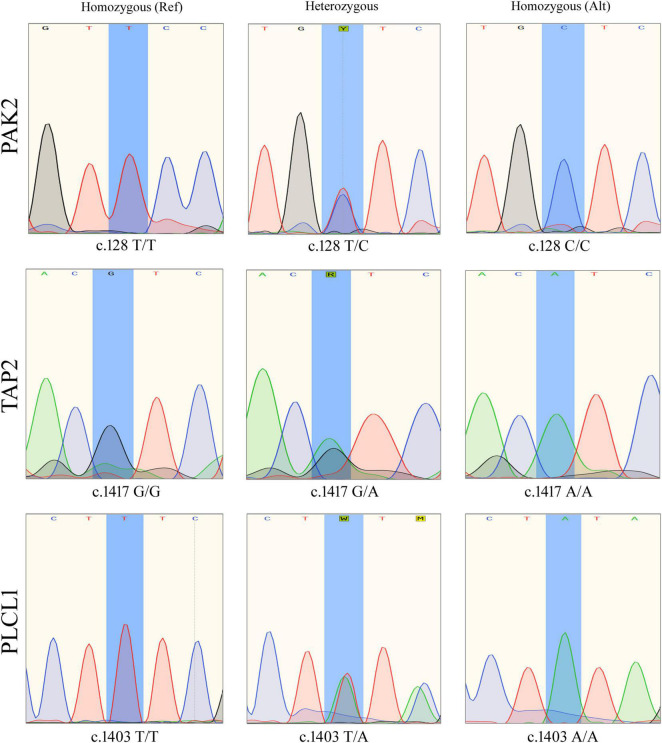
Sanger sequencing results showing different genotypes of *PAK2* (c.128T>C), *TAP2* (c.1417G>A), and *PLCL1* (c.1403T>A) variants.

Sanger sequencing has confirmed that all affected individuals (IV.5, V.1 and V.2) have all the three variants together (*PAK2* (c.128T > C, p. V43A), *TAP2* (c.1417G > A, p. F468Y), and *PLCL1* (c.1403T > A, p. V473I), while healthy individuals, including the father, have only one or two of them (Figure 2).

### Computational Functional Analysis

#### Protein-Protein Interaction and Pathways Enrichment Analysis

**FIGURE 3:**
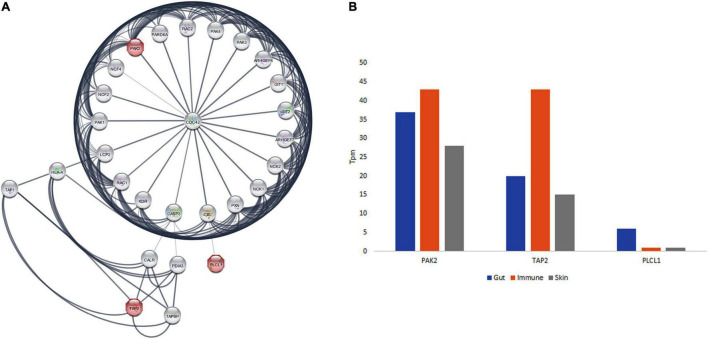
The protein interaction network of *PAK2, TAP2*, and *PLCL1* and GTEx database expression analysis in gut, immune and skin.

PPI network analysis for *PAK2*, *TAP2*, and *PLCL1* revealed that there are 25 nodes with 133 edges, which is significantly more than the expected number of edges (*p* < 1.0e^–16^). It was found that *PAK2*, *TAP2*, and *PLCL1* are directly interacting with 25 proteins each. Pathway enrichment analysis has revealed that 38 KEGG, 46 Reactome, and 49 Wiki pathways were significantly enriched. Among the pathways, the signaling by receptor tyrosine kinases (*PAK2*; *p* = 2.69e^–20^), T-cell receptor signaling (*PAK2*-*PLCL1*; *p* = 5.37e^–10^), GABA and human immunodeficiency pathways (*PAK2*-*TAP2*; *p* = 1.13e^–06^) are significantly enriched (Figure 3A).

#### Gene Ontology Analysis

The GO analysis of *PAK2*, *TAP2*, and *PLCL1* genes showed their involvement in 106 GO terms, in the cellular components (22 GO terms), biological processes (48 GO terms), and molecular function (36 GO terms) categories. All the annotations together highlight that these three genes are localized to the membrane and plasma membrane (cellular component) and play a significant role in the adaptive immune system, immune system process, and signal transduction (biological process), as well as ATP and MHC binding and GABA receptor binding function. The *PAK2* gene plays an important role in the T cell receptor signaling pathway, human immunodeficiency, and signaling by receptor tyrosine kinases. *TAP2* is associated with Antigen processing and presentation, and primary immunodeficiency, and *PLCL1 in* GABAergic synapse, and T cell receptor signaling pathway (Figure 3A).

#### RNA Expression Analysis

From the GTEx data, the positive expression state of *PAK2*, *TAP2*, and *PLCL1* genes in various gut tissues (colon, duodenum, large intestine, rectum, sigmoid colon, small intestine, small intestine Peyer’s patch, transverse colon, vermiform appendix, transverse colon, and vermiform appendix), immune function related organs (leukocyte, spleen, lymph node, thymus, EBV-transformed lymphocyte, bone marrow, and small intestinal Peyer’s patches), and skin of the body (lower leg skin, skin, suprapubic skin, and zone of skin) was observed. Moreover, the highest expression was seen in the immune function-related organs for *PAK2* and *TAP2* genes. The *PLCL1* gene showed the highest expression in the gut tissues (Figure 3B).

### Computational Sequence and Structural Analysis

#### Pathogenic Predictions and Conservation

**FIGURE 4:**
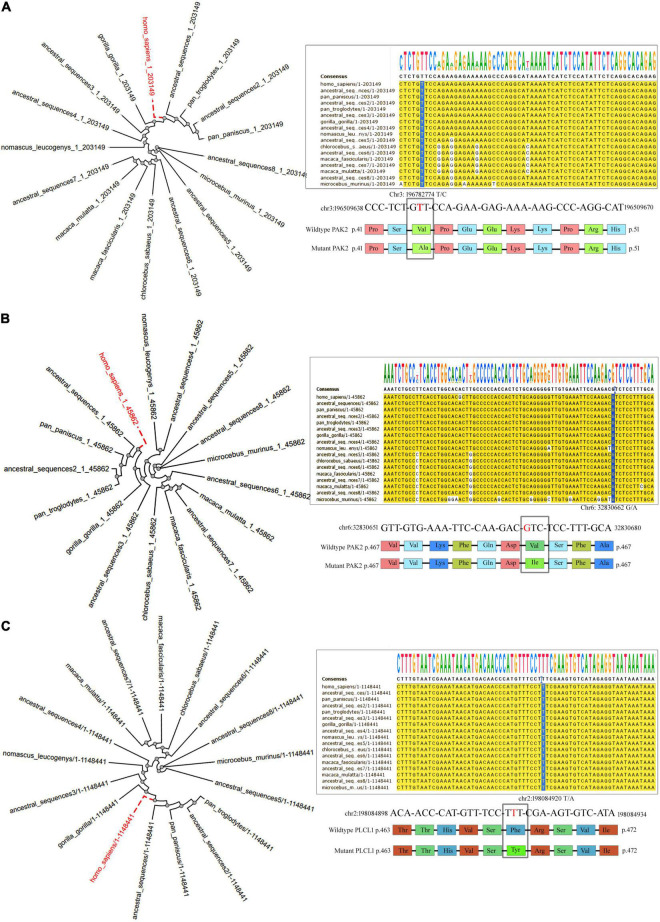
The Phylogenetic tree and nucleotide sequence alignment showing the location of missense variants of the *PAK2* **(A)**, *TAP2* **(B)**, and *PLCL1* **(C)** genes across 13 primates.

Variants in *TAP2* and *PLCL1* appeared to have an allele frequency of 0.0106 and 0.0098 in SHGP and GME (The Greater Middle East) databases. Only one individual had a homozygous *PLCL1*, and 12 individuals had a homozygous *TAP2* variant. But the *PAK2* variant is not seen in both SHGP and GME. The gnomAD showed that the variants in *TAP2* and *PLCL1* have a MAF of 2.398e^–05^ and 4.46e^–05^, respectively. The *PAK2* variant is also not seen in gnomAD. Pathogenicity prediction of *PAK2* (V43A), *TAP2* (F468Y) and *PLCL1* (V473I) variants is deleterious. Conserved domain analysis has shown that the human *PLCL1, PAK2* and *TAP2* proteins have 4, 2 and 1 functional domains, respectively. Sequence conservation pattern suggests that all the identified variants were located in evolutionarily conserved region (Figure 4). Phylogenetic analysis among closely related species (12 selected primates) revealed that the variants in three genes of interest showed a significantly close phylogenetic relationship with Bonobo and Chimpanzee but deviated from Mouse Lemur (Figure 4).

#### The Secondary and 3D Structure Prediction and Analysis

**FIGURE 5:**
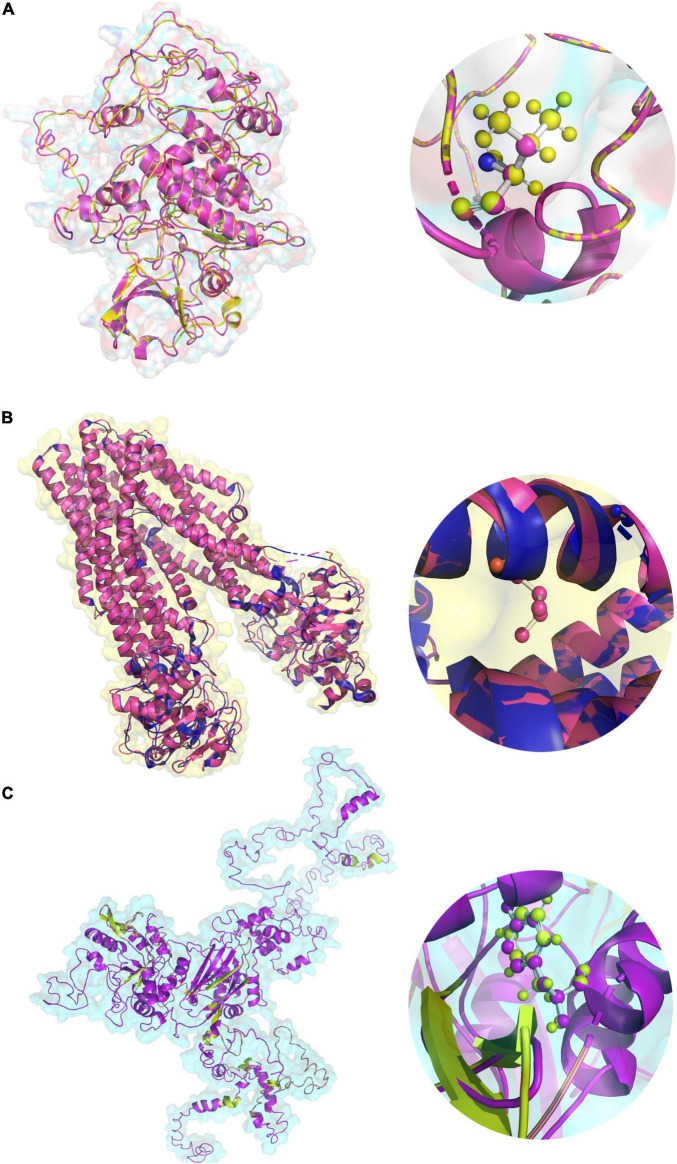
The 3D protein structures of **(A)** *PAK2* (V43A), **(B)** *TAP2* (F468Y), **(C)** *PLCL1* (V473I) in both native and variant forms.

The V43A variant of the *PAK2* gene was mapped 30 amino acids downstream to the P21-Rho-binding functional domain. However, the solvent accessibility analysis suggests that the V43A variant increases the residue access to solvent. In contrast, the F468Y variant of the *TAP2* gene and the V473I variant of the *PLCL1* gene are localized in ATPases associated with a variety of cellular activities (495–678) domain and Phospholipase C, catalytic domain X (398–542) domain, respectively. Solvent accessibility analysis shows that the F468Y variant of the *TAP2* gene has no effect, while the V473I variant of the *PLCL1* gene increases residue access. Furthermore, to understand the effect of variants on the 3D structure of the protein, we performed homology modeling. The human *TAP2* protein structure (5U1D), from the PDB, was collected for further analysis. To build the *PLCL1* and *PAK2* protein models, Blast-p against PDB was carried out to identify the suitable template for structure generation (1DJG, 1QAS, 5UPL, and 4ZJ1) by I-Tasser. After a successful build of a protein structure, it was energy minimized and validated. Maximum numbers of amino acid residues fall into the highly preferred region of the Ramachandran plot. All the wild-type protein structures were then used to build the corresponding mutant protein structures using the DUET web server (Figure 5). The integrated computational approach predicted that the identified rare substitutions of *PAK2* (ΔΔG is -0.241 kcal/mol), *PLCL1* (G is -1.302 kcal/mol), and *TAP2* (G is -0.68 kcal/mol) significantly destabilize their structures and functions (Table 3).

**TABLE 3:** Protein sequence annotation and structural stability prediction results for *PAK2, TAP2*, and *PLCL1.*

	Gene	*PAK2*	*TAP2*	*PLCL1*
Protein sequence annotation	Variant	V43A	F468Y	V473I
	Solvent accessibility	Increased	No effect	Increased
Protein structure stability prediction	mCSM	–0.386 kcal/mol (Destabilizing)	–0.191 kcal/mol (Destabilizing)	−1.391 kcal/mol (Destabilizing)
	SDM	−0.75 kcal/mol (Destabilizing)	−0.68 kcal/mol (destabilizing)	−0.86 kcal/mol (Destabilizing)
	DUET	–0.241 kcal/mol (Destabilizing)	0.077 kcal/mol (Stabilize)	−1.302 kcal/mol (Destabilizing)

## Discussion

The co-existence of multiple autoimmune diseases has been described using the general term “the kaleidoscope of autoimmunity.” This term includes the possibility of the alteration from one AiD to another AiD and the co-existence of multiple AiDs in one patient or within the family (familial aggregation) (31, 32). Many autoimmune diseases were reported together in one individual, especially with type 1 diabetes mellitus (35%), systemic lupus erythematosus (41%), primary biliary cirrhosis (32%), Sjögren’s syndrome (33%), autoimmune thyroid diseases (15%), antiphospholipid syndrome (28%), vitiligo (27%), systemic sclerosis (26%), multiple sclerosis (15%), rheumatoid arthritis (15%), myasthenia gravis (13%), and alopecia areata (10%) (33). One such case was reported of a young woman with seven autoimmune diseases together: rheumatoid arthritis (RA), idiopathic thrombocytopenic purpura, pernicious anemia, Hashimoto’s thyroiditis, systemic sclerosis (SSc), pancreatic exocrine insufficiency, and celiac disease (31, 34). Familial aggregation of many autoimmune diseases in consanguineous families will be ideal to explore the shared causal gene/s for multiple autoimmune diseases. We analyzed such a family with four different diseases seen in three affected members of the family.

We analyzed WES data of a rare consanguineous Saudi family with four auto-immune diseases (CeD, AiT, SLE, and TIDM) affecting three people: the mother and two of her children. The main objective of this study is to scan for rare deleterious variants that could explain the common pathways and genes playing a critical role in this family with the inheritance of the 4 autoimmune diseases: SLE, T1DM, Thyroid, and CeD. We identified rare variants in *PAK2*, *TAP2*, and *PLCL1* genes which have been reported previously to be functionally relevant to CeD, AiT, SLE, and TIDM (35–37). These three genes are located at 3q29, 6p21.32, and 2q33.1, respectively. In this family, affected individuals inherited at least one of the variants as homozygotes and the rest as heterozygotes. None of the healthy relatives are homozygotes for any of these variants. We have provided the strongest evidence for three non-MHC genes that were previously associated with other autoimmune diseases and might be the key players in the etiology of these diseases among susceptible individuals. We suggest that complex interaction and inheritance of rare variants in three genes in this consanguineous family results in four autoimmune diseases affecting multiple organs.

In SHGP and the GME databases, all the three variants are very rare, especially in a homozygote condition. The frequency of the variant of *PLCL1* in the SHGP, GME, and gnomAD databases is 0.0004 and 0.0025 and 2.398e^–05^, respectively. The *PAK2* variant is not observed in the GME, SHGP, or genomAD databases. The *TAP2* variant MAF is 0.002, 0.0016, and 4.461e^–05^ in SHGP GME and gnomAD, respectively. Putting these frequencies together, the probability of the coexistence of these three variants in one individual among the Saudi population is rare with a frequency approximately 0.0000008, and 0.000004 in the population. The presence of rare variants in these genes might play a crucial role in the development of the autoimmune diseases due to their complex interactions in key related pathways regulating receptor binding and FCGR3A-mediated IL10 synthesis, which is an important immunoregulatory cytokine.

*PLCL1* and *PAK2* genes are essential for signal transduction and cellular regulation, including cell motility, cytoskeletal dynamics, gene transcription, cell cycle progression, and death and survival signaling. The membrane-associated protein, *TAP2*, is one of the ATP-binding cassette (ABC) transporters. This protein is a member of the MDR/TAP subfamily, involved in multidrug resistance. A study conducted on families with three different autoimmune diseases (SLE, Sjögren’s syndrome, and rheumatoid arthritis) identified 39 rare variants in immune-related genes, including TCR signaling pathway genes, especially *PAK2* and *PLCL1*, signifying the role of a complex interaction of multiple genes in families with autoimmune diseases (37). Another family-based study illustrated a significant association between *TAP2* and SLE disease (35). Furthermore, the *TAP2* gene is also associated with both T1DM and AiT (36).

Interestingly, the PPI demonstrated that *PLCL1* interacts with E3 ubiquitin-protein ligase CBL (CBL). CBL is functionally connected with HLA-A and HLA-DQA, which is a significant risk factor for Celiac disease. The HLA genes are also functional interacting partners of the *TAP2* gene, involved in the presentation of foreign antigens to the immune system. Furthermore, CBL is an essential protein that triggers the threshold for T cell activation and regulates peripheral T cell tolerance through a variety of processes. It also controls innate immune responses and immune activations through functioning as a negative controller of immune activations and plays a crucial role in host defense against pathogens (38, 39). On the other hand, *PAK2* interacting partners such as *ARHGEF7*, also known as *PAK3* (a positive regulator of apoptosis) and Lymphocyte cytosolic protein 2 (*LCP2*), have been previously linked to human immunodeficiency (40). These are also known to have functional connectivity with CBL (41), by influencing or interacting with *PLCL1* through the phosphatidylinositol-specific phospholipase C, X domain.

We propose that CeD, AiT, SLE, and TIDM could be influenced by different genes through rare variants which dysregulate their functions in a shared pathway or indirectly linked pathways. For the first time, such co-inheritance of rare variants was found in *PLCL1*, *PAK2*, and *TAP2* genes on three different chromosomes in a single consanguineous family, dysregulating the TCR signaling and the antigen processing pathways. Thus, this might trigger the CD4 + T cells to produce different subsets of helper T cells. An important role for Th-cell activation is described by cytokine secretion, involving Th1, Th2, and Th17, which are major contributors to CeD, AiT, SLE, and TIDM pathogenesis (42–45).

There are some limitations to the current study that we acknowledge. Our study was carried out on one rare consanguineous family with the aggregation of multi-autoimmune diseases. Recruiting more such families may enable us to identify the crucial genes and pathways involved in the familial aggregation of multi-autoimmune diseases.

## Conclusion

This study has identified the trigenic inheritance of rare missense variants in *PAK2* (c.128T > C, p. Val43Ala), *TAP2* (c.1417G > A, p. Val473Ile), and *PLCL1* (c.1403T > A, p. Phe468Tyr) genes as the molecular basis of polygenic autoimmune diseases CeD, AiT, SLE, and TIDM. We propose that alternate explanations should be explored, in cases, where classical inheritance models fail to explain the genetic basis of autoimmune diseases. The rare variants we identified in the *PAK2*, *TAP2*, p. Val473Ile, and *PLCL1* genes are postulated to contribute to the pathogenesis of CeD, AiT, SLE, and TIDM by modulating antigen processing and presentation, T cell receptor signaling, and immunodeficiency pathways. It is worth mentioning the importance of functional analysis in the animal and *in vitro* models to understand the involvement of these genes and the role of these variants in unraveling the complex molecular interactions of multiple genes in autoimmune disorders.

## Data Availability Statement

The datasets presented in this article are not readily available because (a) participants’ refusal to store or distribute the genomic data in the public domain and (b) as per the local Institutional Ethics Committee approval and national policy on genomic data sharing in the public domain outside the country. Allowed data under the above-mentioned restrictions of the IRB and participants’ requirements are presented in the article as well in the Supplementary Material, further inquiries can be directed to the corresponding author/s.

## Ethics Statement

The studies involving human participants were reviewed and approved by Institutional Review Board (IRB) at King Abdulaziz University Hospital. Written informed consent to participate in this study was provided by the participants’ legal guardian/next of kin.

## Author Contributions

BB, RE, and NS: conceptualization. AA, BB, NS, and RE: methodology and writing – original draft preparation. AA and BB: software and visualization. AA, BB, and NS: formal analysis. AA, OS, BB, RE, BA-S, and NS: investigation. BB: resources. AA, SH, OR, MA, BA-S, HA, YB-T, BB, OS, RE, KA, KN, and NS: writing – review and editing. SH, BB, NS, and RE: supervision. NS: project administration and funding acquisition. All authors contributed to the article and approved the submitted version.

## Conflict of Interest

The authors declare that the research was constructed in the absence of any commercial or financial relationships that could be construed as a potential conflict of interest.

## Publisher’s Note

All claims expressed in this article are solely those of the authors and do not necessarily represent those of their affiliated organizations, or those of the publisher, the editors and the reviewers. Any product that may be evaluated in this article, or claim that may be made by its manufacturer, is not guaranteed or endorsed by the publisher.

## Footnotes

1
http://bio-bwa.sourceforge.net/

2
https://www.saudigenomeproject.org/en/

3
http://www.mbio.ncsu.edu/

4
https://string-db.org/

5
https://www.ensembl.org/index.html

6
http://smart.embl-heidelberg.de/

7
www.esembl.org

8
https://ngphylogeny.fr/

9
https://zlab.umassmed.edu/bu/rama/

10
http://biosig.unimelb.edu.au/duet/

11
https://predictprotein.org
