# Supplementary material for: Complex Inheritance of Rare Missense Variants in PAK2, TAP2, and PLCL1 Genes in a Consanguineous Arab Family With Multiple Autoimmune Diseases Including Celiac Disease
Source: Front Pediatr. 2022 Jun 15;10:895298. doi: 10.3389/fped.2022.895298 (PMC9242504; doi:10.3389/fped.2022.895298)
Supplement: Supplementary file 1 [file Data_Sheet_1.docx]

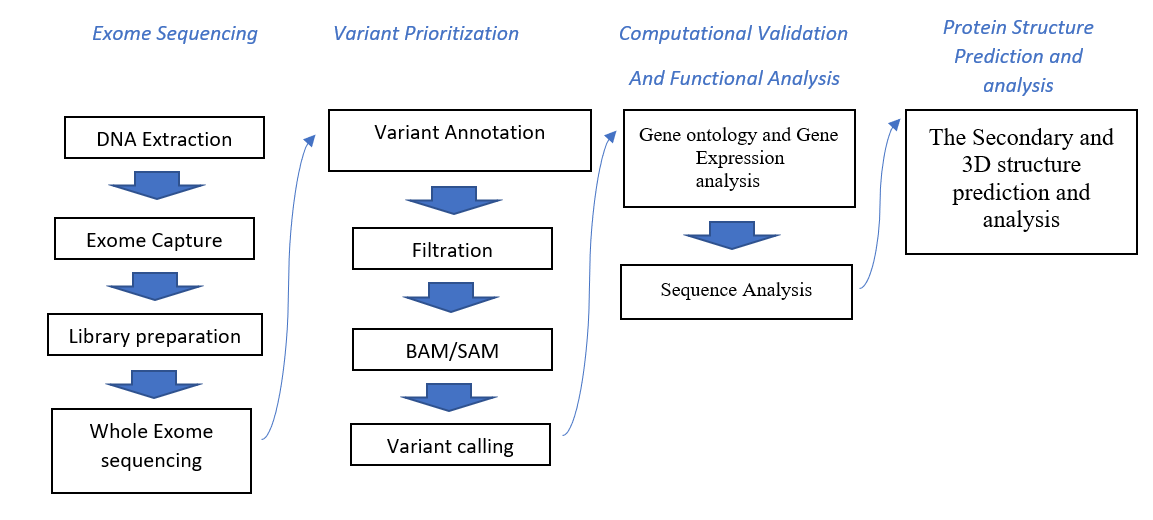


Supplementary Figure S1: Overall workflow of the current study


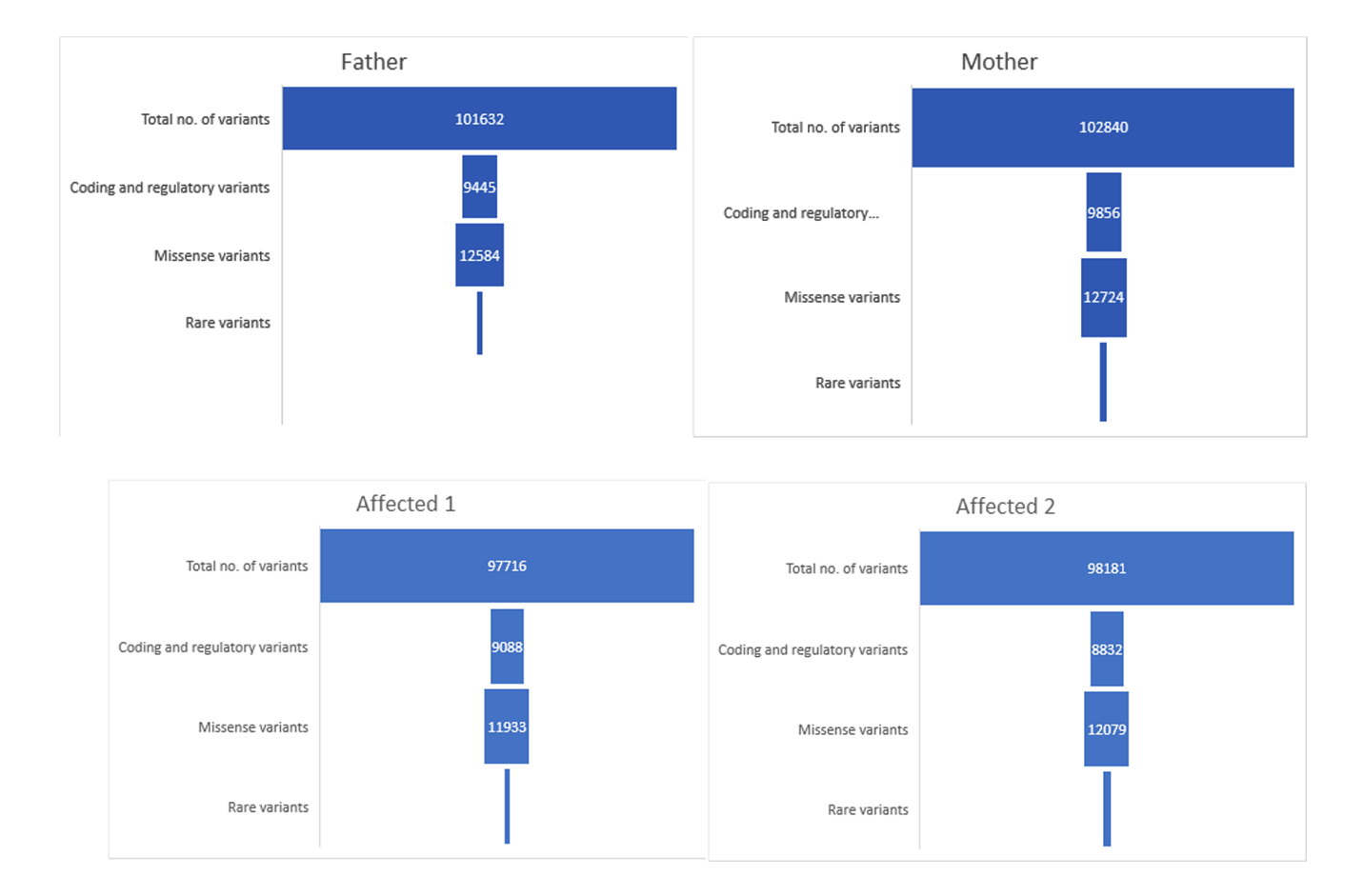


Supplementary Figure S2: Variant filtration steps in exome analysis
